# Artificial Intelligence in Medical Writing: Is It an Exception to Evidence-Based Medicine?

**DOI:** 10.31662/jmaj.2025-0443

**Published:** 2025-12-05

**Authors:** Shigeki Matsubara

**Affiliations:** 1 Department of Obstetrics and Gynecology, Jichi Medical University, Tochigi, Japan; 2 Department of Obstetrics and Gynecology, Koga Red Cross Hospital, Koga, Ibaraki, Japan; 3 Medical Examination Center, Ibaraki Western Medical Center, Chikusei, Ibaraki, Japan

**Keywords:** artificial intelligence, ChatGPT, evidence-based medicine, future, regulation

## Abstract

Generative artificial intelligence (GenAI) is now widely used in medicine, including medical writing. Its merits and demerits have been discussed; however, such discussion has not been based on evidence-based medicine (EBM). Here, I focus primarily on GenAI use in medical writing, illustrating how it has already spread before its safety―especially long-term safety―has been confirmed by EBM. I therefore make several modest proposals. Assuming GenAI is a new drug, its use has not yet cleared even the first step of a phase I trial. Assuming it is a new procedure, it remains at the “experience” or “case report” phase. EBM requires the completion of phase I-III trials and randomized controlled trials or meta-analyses before any drug or procedure is confirmed safe and effective. Emergency evacuation can be applied for life-threatening medical conditions; however, it does not apply to “writing.” Nevertheless, the current publication world has already gone far beyond: GenAI use is already considerable in medical publication. Thus, three propositions have been made. First, we must recognize that the use of GenAI for writing operates outside the usual EBM framework. Second, we should conduct trials, even if they are difficult and time-consuming, to evaluate the safety and effectiveness of GenAI in writing. Third, we should use GenAI in writing only modestly until safety is confirmed. What is true becomes evident long after, and thus, I believe that we should take a cautious stance toward GenAI use in writing. How cautious should be discussed widely. This viewpoint may contribute to the discussion of GenAI use more generally, beyond medical writing.

## Worldwide Concern about Generative Artificial Intelligence (GenAI) Use in Medical Writing

The public emergence of ChatGPT has generated broad discussion about its positive and negative effects on medicine in general, particularly on medical writing. Here, I primarily describe aspects of GenAI (such as ChatGPT) use in medical writing while briefly touching on its use in medicine more broadly. For simplicity, I will refer to GenAI simply as ChatGPT throughout this text. Of many positive and negative effects, the following represent key examples. ChatGPT may streamline medical writing and save time for authors ^(1), (2)^, which is considered especially beneficial for non-English-speaking researchers―this is a positive effect. In contrast, ChatGPT may dilute the human tone and individual thinking of authors; it may also push many to write hastily, thereby reinforcing a “publish and perish” culture without contributing to real medical progress ^(1)^. It can also benefit publishers that favor open access with article-processing charges, thereby distorting the publication landscape―this is a negative effect ^(1)^.

## My Concern about ChatGPT Use in Medical Writing

Although I acknowledge the potential merits of ChatGPT in medical writing, I am concerned that its use may cause serious problems for authors, primarily researchers and clinicians. My primary concern is straightforward. Although the safety of ChatGPT in medical writing, especially its safety for the future, has not yet been established, the publication world has already moved far ahead, adopting these tools without clear evaluation. Indeed, a bibliometric analysis of GenAI regulations in medical journals showed considerable variation in the permissible extent of GenAI use among top journals ^(2)^. As an extreme example, some journals have recently declared that GenAI use in writing is openly allowed without disclosure ^(3)^. Importantly, these journal regulations are not based on any global, inter-journal discussion of GenAI’s safety, particularly its future safety ^(2), (3)^. Later, I explain how evidence supporting ChatGPT use in writing remains immature, using evidence-based medicine (EBM) as an analogy.

## ChatGPT Use in Writing Has Not Passed the Usual EBM Procedures

ChatGPT appears to be used without following the standard procedures we have long respected. First, let us consider ChatGPT as a new “drug.” ChatGPT may save time for busy physician-researchers and even produce more appealing manuscripts than many human authors. However, before any new drug is approved, clinical trials are mandatory: phase I (safety), phase II (effectiveness), and phase III (large-scale confirmation). If a drug fails at phase I―that is, if safety is not guaranteed―the trial is immediately stopped, and the drug is not introduced. Safety always outweighs effectiveness. I believe that possible harms of ChatGPT include i) deterioration of human writing ability, ii) dilution of individual voice and homogenization of medical articles, and iii) creation of digital divides, leading to inequity. Although merits of ChatGPT use in writing may exist, we must remember: safety should always take precedence over effectiveness.

Second, let us consider ChatGPT as a new medical technology, such as a novel surgical technique, if the drug analogy seems extreme. The pathway to its standard use in medical practice is fundamentally similar, following established protocols. A new technology may first be tried through personal experience, then reported as case reports, followed by observational studies, randomized controlled trials (RCTs), and finally meta-analyses. Only after this sequence―that is, on the basis of EBM―do we accept a new procedure as safe and effective. At present, ChatGPT use in medical writing is at best at the “experience” or “case report or observational study” stage, far from phase I confirmation or RCTs, let alone phase III or meta-analyses in terms of EBM.

## “Emergency Evacuation” Does Not Fit for “Writing”

We must distinguish ChatGPT use in writing from its application in medical practice. If a treatment is urgently needed, regulators may allow drugs or techniques under special rules, sometimes called an “emergency evacuation.” However, I am not referring to lifesaving diagnosis or treatment; I am referring to writing. I cannot imagine any situation in which the use of ChatGPT for writing should be accepted under emergency evacuation rules.

## ChatGPT Is Already Used in Publication

We must look at the current situation: GenAI has already entered the publication world. One study using the artificial intelligence (AI) detector ZeroGPT found that 16.7% of 3,374 post-AI-era orthopedic studies exceeded the detection threshold ^(4)^. Some journals that have allowed unrestricted GenAI use in manuscript writing have done so, also considering the wide and inevitable usage of ChatGPT in medical publications ^(3)^. Similar views are expressed elsewhere ^(2)^. GenAI is already present, and its use is increasing.

## How Should We Consider the Relationship between ChatGPT and EBM?

This situation raises an important question: how should we consider the relationship between ChatGPT use in writing and EBM? My concern is not with today’s hybrid writing, in which authors still understand their own writing, whereas some are partly assisted by GenAI. Rather, it is with the next generation, who may never, or barely, learn to write independently. GenAI risks hollowing out writing itself, a deeply human cognitive act. In ordinary medicine, many years or even decades are required before a new drug or surgical technique passes through trials and meta-analyses confirming both safety and effectiveness. The impact of ChatGPT on human cognition can only be observed in the future, and this observation may require even longer than the usual period needed in ordinary medicine. Thus, “observe and think longer” is theoretically an even more extended process when it comes to ChatGPT use. Must we wait until then, until EBM provides guidance?

Theoretically, we should wait for meta-analyses before deciding how to use ChatGPT in medical writing. This approach follows the principles of EBM that we have long respected. However, we must also confront the reality of the present situation.

## My Three Propositions

I offer three simple propositions. First, we must recognize that the use of ChatGPT for writing operates outside the usual EBM framework. This represents an exceptional scenario in medical progress. Second, we should conduct trials―even if they are difficult and time-consuming―to evaluate the safety and effectiveness of ChatGPT in writing. Third, because such evidence will not be available for many years, we should use ChatGPT only modestly until safety is confirmed. The medical publication community must discuss and define what “modestly” actually means.

## What Is True Becomes Evident Long After, and Thus, I Ask the Medical Publication World

I am concerned about the future and believe we must prepare, at least in mindset, for the worst. GenAI, I believe, represents a Copernican revolution in medical writing. The Nobel Prize, usually awarded to researchers who have brought about such a revolution, is often given decades after the initial discovery; time is needed to confirm a true revolution. Imagine that in 2050, meta-analyses conclude that physicians’ writing and thinking skills have markedly deteriorated compared with pre-AI levels. What then? Will we simply shrug―“well, that’s life”―and accept the loss, or belatedly attempt to prohibit AI use? This scenario is not mere fiction but a theoretical possibility. We must prepare ourselves, at least mentally, to face such a potential outcome.

## Widening the View beyond Medical Writing

In this article, I primarily described ChatGPT use in medical writing and emphasized that its adoption is not based on standard medical practice such as EBM. Broadening the view, AI is also used widely in medicine beyond writing. Covering AI in medicine generally is beyond the present scope, yet we must consider each area separately―AI in diagnosis, treatment, shared decision-making, and others. A one-size-fits-all discussion is impossible. However, I believe that some points discussed here may inform the broader consideration of AI in medicine. An important argument has been made that AI in medicine should not be treated merely as a technical matter but as something that must preserve human values ^(5)^; I strongly agree. Similarly, writing is not a purely technical act but an expression of individual human tone, taste, and thought. Preserving this human value will, in the long run, support genuine medical progress.

## When I Calmly Reflect…

I calmly reflect on the situation. The possibility that something is going wrong is difficult to deny completely. In such cases, it is wiser to err on the side of caution. My career as a writer is drawing to a close. Nevertheless, I still care deeply about the world of medical writing, where I have lived and which I have quietly cherished for half a century.

## Article Information

### Author Contributions

Shigeki Matsubara identified the significance and wrote the manuscript. Shigeki Matsubara met the ICMJE guidelines for authorship, approved the submitted manuscript, and agreed to be accountable for all aspects of the work.

### Conflicts of Interest

None

### Approval of Institutional Review Board

Not applicable.

### Patient Anonymity

Not applicable.

### Informed Consent

Not applicable.

### Data Availability Statement

Data sharing is not applicable to this article because no new data were created or analyzed in this study.
